# Prevention of venous thromboembolism in right heart–sided electrophysiological procedures: results of an European Heart Rhythm Association survey

**DOI:** 10.1093/europace/euad364

**Published:** 2023-12-13

**Authors:** Giacomo Mugnai, Michal Farkowski, Luca Tomasi, Laurent Roten, Federico Migliore, Carlo de Asmundis, Giulio Conte, Serge Boveda, Julian K R Chun

**Affiliations:** Division of Cardiology, Cardio-Thoracic Department, School of Medicine, University Hospital of Verona, Piazzale Aristide Stefani 1, 37126 Verona, Italy; Department of Cardiology, Ministry of Interior and Administration National Medical Institute, Warsaw, Poland; Division of Cardiology, Cardio-Thoracic Department, School of Medicine, University Hospital of Verona, Piazzale Aristide Stefani 1, 37126 Verona, Italy; Department of Cardiology, Inselspital, Bern University Hospital, University of Bern, Bern, Switzerland; Department of Cardiac, Thoracic, Vascular Sciences and Public Health, University Hospital of Padova, Padova, Italy; Heart Rhythm Management Centre, Universitair Ziekenhuis Brussel, Postgraduate Program in Cardiac Electrophysiology and Pacing, European Reference Networks Guard-Heart, Vrije Universiteit Brussel, Brussels, Belgium; Division of Cardiology, Cardiocentro Ticino, Lugano, Switzerland; Heart Rhythm Management Department, Clinique Pasteur, Toulouse, France; Cardioangiologisches Centrum Bethanien (CCB), Medizinische Klinik III, Agaplesion Markus Krankenhaus, Frankfurt Am Main, Germany

**Keywords:** Catheter ablation, Electrophysiological study, Right-sided ablations, Anticoagulation

## Abstract

Limited data are available regarding venous thromboembolism (VTE), specifically deep vein thrombosis (DVT) and pulmonary embolism (PE), following right-sided ablations and electrophysiological (EP) studies. Compared to left-sided procedures, no guidelines on antithrombotic management strategies for the prevention of DVT and PE are available. The main purpose of the present European Heart Rhythm Association (EHRA) survey is to report the current management of right-sided EP procedures, focusing on anticoagulation and prevention of VTE. An online survey was conducted using the EHRA infrastructure. A total of 244 participants answered a 19-items questionnaire on the periprocedural management of EP studies and right-sided catheter ablations. The right femoral vein is the most common access for EP studies and right-sided procedures. An ultrasound-guided approach is employed by more than 2/3 of respondents. Intravenous heparin is not commonly given by the majority of participants. About 1/3 of participants (34%) routinely prescribe VTE prophylaxis during (mostly aspirin and low molecular weight heparin) and 1/4 of respondents (25%) commonly prescribe VTE prophylaxis after discharge (mostly aspirin). Of note, respectively 13% and 9% of participants observed at least one DVT and one PE related to right-sided ablation or EP study within the last year in their center. The present survey shows that only a minority of operators routinely gives intraprocedural intravenous heparin and prescribes VTE prophylaxis after right-sided EP procedures. Compared to left-sided procedures like atrial fibrillation (AF) ablation, there are no consistent systematic antithrombotic management strategies.

## Graphical Abstract

**Graphical Abstract:**
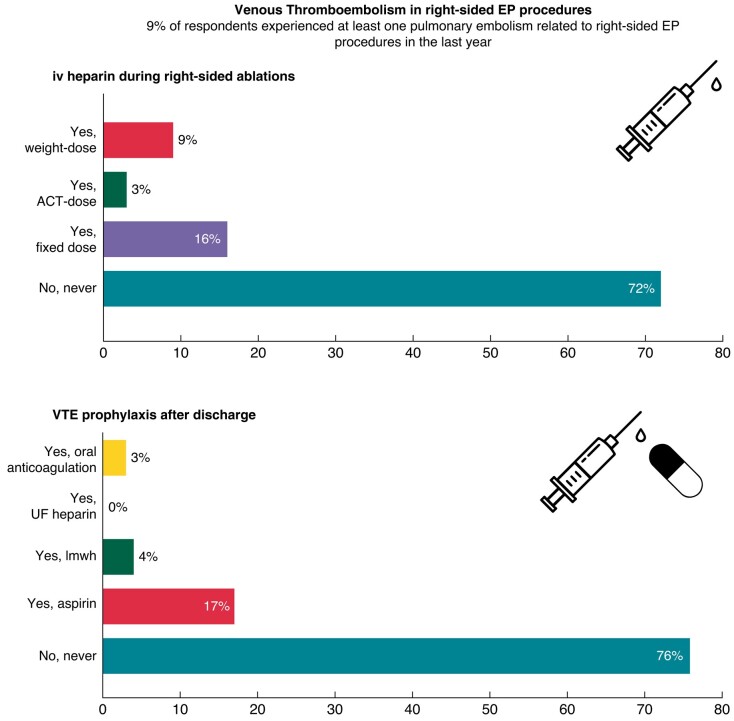
EP, electrophysiological; ACT, activated clotting time; iv., intravenous; VTE, venous thromboembolism; UF, unfractionated; lmwh, low-molecular-weight heparin.

**What’s new?**

- The echo-guided approach is currently adopted in a routine way by only one-third of respondents (37%).
- The ‘heparin bridging’ in patients on vitamin K antagonist is still adopted by a significant amount of physicians (29%).
- Intravenous heparin is adopted by a smaller but not negligible amount of European electrophysiologists during electrophysiological (EP) studies (28%) and right-sided ablation (37%).
- Venous thromboembolism (VTE) prophylaxis is—on average—adopted by one operator of four after the discharge (25%), and the type of VTE prophylaxis is heterogeneous.
- Among operators who prescribe VTE prophylaxis, the majority (71%) gives aspirin.
- No significant differences were found in anticoagulation strategies neither between Northern and Southern Europe nor between fully trained electrophysiologists and senior/junior EP fellows.

## Introduction

Femoral venous access is commonly used for all right-sided ablation procedures and for diagnostic electrophysiological (EP) studies. Limited data are available about venous thromboembolism (VTE), specifically deep vein thrombosis (DVT) and pulmonary embolism (PE), following right-sided ablations and EP studies^1–13^ In left-sided ablation procedures, especially pulmonary vein isolation, the peri-procedural approach is well standardized and homogenous throughout most of the centres and guidelines exist.^14,15^ Conversely, for right-sided procedures, there is no clear guidance from guidelines and not broadly accepted or standardized approach/workflow. Symptomatic DVT and PE following right-sided EP procedures are globally reported in 0.5–0.8% of patients.^1^ Although VTE is considered a rare event, it might potentially lead to severe consequences. The main purpose of this European Heart Rhythm Association (EHRA) survey was to investigate the management of right-sided ablations, especially in terms of anticoagulation and prevention of VTE, among cardiac electrophysiologists.

## Methods

This physician-based survey was promoted and disseminated by EHRA. An online questionnaire, consisting of 19 multiple choice questions, was developed, amended, and validated by the Scientific Initiative Committee (SIC).

The questionnaire was distributed through official EHRA channels (EHRA newsletters, Scientific Research Network members, and national electrophysiology working groups) and social media platforms. It was active between 18 October and 21 November 2022. All cardiologists working in the field of Electrophysiology, from junior cardiac electrophysiology fellows to senior fellows until to fully trained cardiac electrophysiologists, were deemed to be eligible to participate in the survey.

In detail, the questionnaire was created by the promotor and then shared with the members of the working group; then, the questionnaire was shared, discussed, and debated with all members of the SIC Committee. The specific queries included in the questionnaire are shown in Supplementary material online.

Results were collected anonymously in compliance with the European General Data Protection Regulation 2016/679.

Data were analysed using descriptive statistical methods. Categorical variables are presented numerically with absolute percentages (%).

### Descriptive statistics

Continuous variables are expressed as mean ± SD or median and range as appropriate. Categorical variables are expressed as absolute and relative frequencies. Comparisons of continuous variables were done with a Student’s *t*-test or the Mann–Whitney *U* test as appropriate. The χ^2^ test or the Fisher’s exact test was used to compare categorical variables as appropriate. A two-tailed probability value of <0.05 was deemed significant. Statistical analyses were conducted using the SPSS software (SPSS v20, Chicago, IL, USA).

## Results

**Figure 1:**
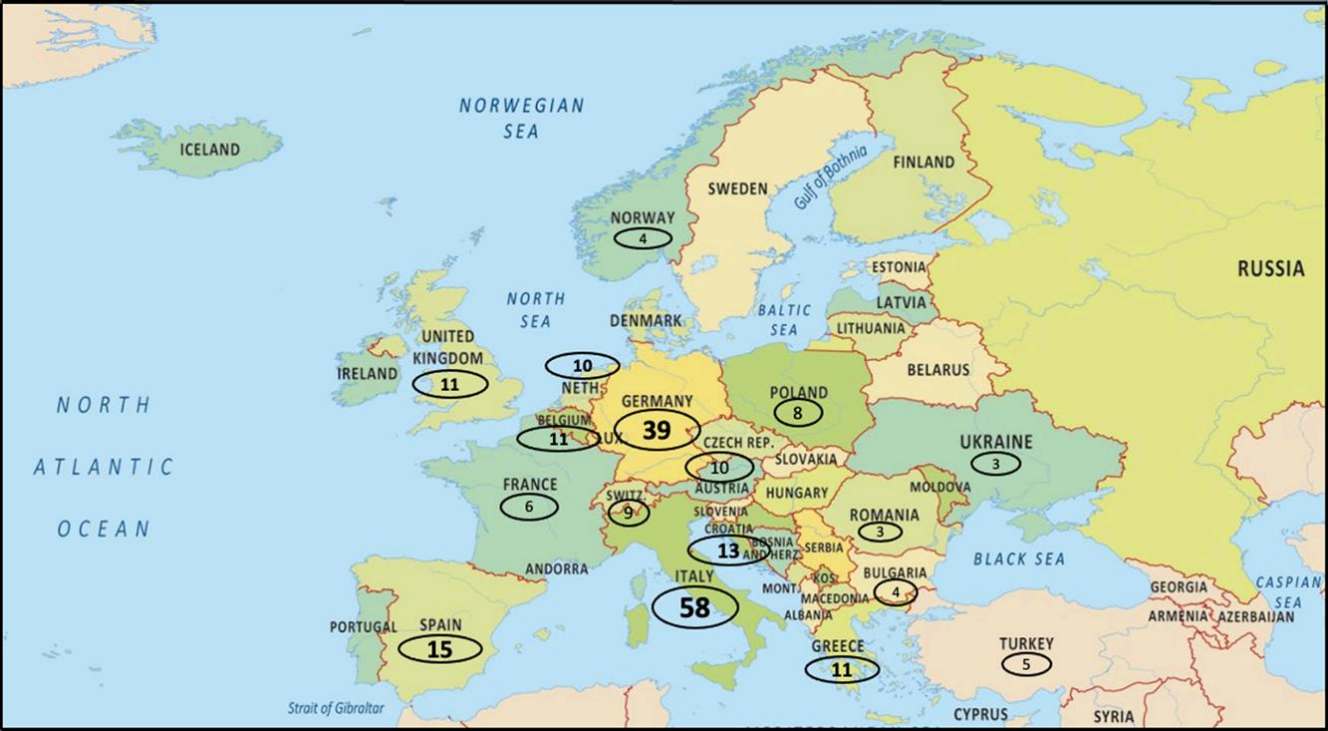
Geographical map showing the distribution of the main responders across Europe.

From 18 October to 21 November 2022, a total of 244 respondents from 33 European countries (Armenia, Austria, Belgium, Belarus, Bulgaria, Croatia, Cyprus, Czech Republic, Denmark, Estonia, France, Georgia, Germany, Greece, Hungary, Iceland, Italy, Latvia, Malta, The Netherlands, Norway, Poland, Portugal, Romania, Russian Federation, Slovakia, Slovenia, Spain, Sweden, Switzerland, Turkey, Ukraine, and UK) participated in the survey (*Figure 1*). Respondents from Italy and Germany most frequently participated in the survey (24 and 16% of the overall participants, respectively) followed by respondents from Spain (6%), Croatia (5%), Belgium (4%), Greece (4%), and UK (4%). The average age of the responders was 43 ± 9 years. The majority of respondents was male (78%).

The majority of participants worked in university hospitals (56%). The remaining participants worked in specialized public cardiology centres (19%), public community hospitals (14%), and private hospitals/clinics (11%). Most of the survey respondents were fully trained cardiac electrophysiologists (73%), followed by junior cardiac electrophysiology fellows (18%) and senior cardiac electrophysiology fellows (9%). The mean number of years of training among participants was 11 ± 8 years [median 10 years, inter-quartile range (IQR) 5–15 years].

The mean volume of right-sided EP procedures performed at the centres of the participants was 166 ± 110/year (median 115/year and IQR 100–215/year). In details, only 4% of the participants performed <50 right-sided procedures per year at their centres; 20% of the respondents worked in centres performing 50–100 right-sided procedures per year; 32% of the physicians declared to perform between 100 and 150 procedures per year; and 10% stated to perform between 150 and 200 procedures. The remaining 34% of participants were active in centres performing more than 200 right-sided EP procedures per year.

Fifty-four percent of respondents stated that, in their centres, they kept a systematic, institutional reporting system used for the collection and assessment of EP procedure-related complications. The median response rate for each question was 80.3% (interquartile range 76.2; 82.3%).

### Venous access and procedural approach

Most of the respondents declared to use two or three catheters (46 and 44%, respectively) for performing EP studies and predominantly three catheters for AVNRT, right accessory pathway, and right AT ablation (55, 57, and 56% of all participants, respectively). Two catheters are most commonly used for right atrial flutter and right ventricular tachycardia/extra-systole ablations (used by 54 and 51% of the overall respondents, respectively); a single catheter approach is mostly used for AV node ablation by 67% of the interviewed sample.

Eighty-one percent of the respondents stated to use only the right groin as venous access (left groin: 0.5%; both right and left groins: 18.5%). The femoral venous access is always obtained by ultrasound-guided approach by 37% of respondents, while, on the contrary, 28% of respondents never use an ultrasound-guided technique for femoral venous puncture. Around one-third of participants (35%) declared to use an ultrasound-guided approach for femoral venous puncture only in selected cases or patients. Most of respondents (84%) never intubate the coronary sinus from the superior jugular/subclavian vein approach, while a small minority (3%) does it in all procedures; of note, 13% of participants cannulate the coronary sinus using a jugular approach only in selected cases, such as accessory pathway ablations (6%), inability to get a femoral access (4%), atrial fibrillation ablation (1%), and AVNRT ablation (1%).

### Anticoagulation management before electrophysiological studies and right-sided ablation procedures

**Figure 2:**
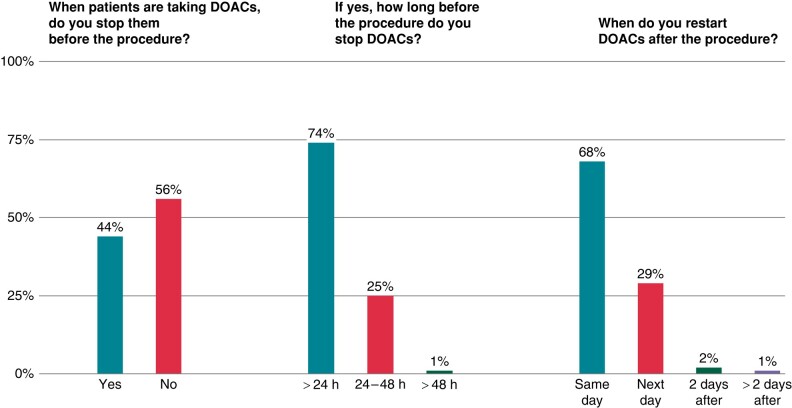
The picture illustrates the management of direct oral anticoagulants before EP studies and right-sided ablation procedures. EP, electrophysiological.

Direct oral anticoagulation (DOAC) is commonly interrupted before EP studies and right-sided ablation procedures by 44% of respondents (*Figure 2*). Among these, 74% stop DOAC therapy within 24 h before the procedure, 25% stop DOAC therapy 24–48 h before the procedure, and 1% suspend the DOAC therapy >48 h before the procedure (*Figure 2*). Among respondents who commonly stop DOAC therapy, the majority (68%) resumes DOAC therapy on the day of the procedure and 29% on the following day (*Figure 2*).

**Figure 3:**
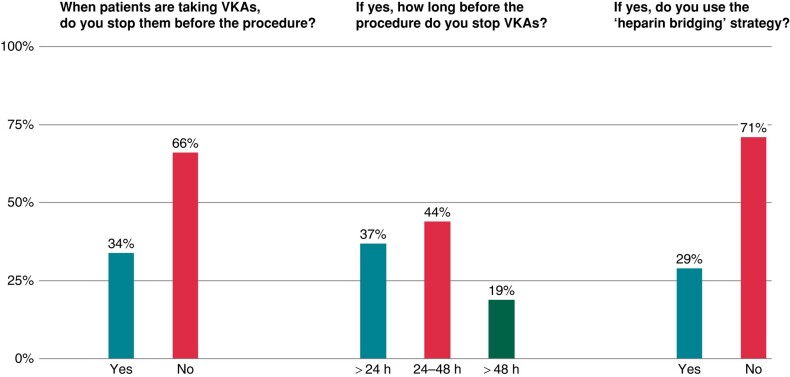
The picture illustrates the management of vitamin K antagonists before EP studies and right-sided ablation procedures. EP, electrophysiological.

On the other side, vitamin K antagonists (VKAs) are interrupted before EP studies and right-sided ablation procedures by 34% of respondents (*Figure 3*). Among the latter, 37% stop VKA therapy within 24 h from the procedure, 44% 24–48 h before the procedure, and 19% suspend VKAs for >48 h before the procedure (*Figure 3*). Of note, the heparin bridging therapy is adopted by 29% of respondents who stop VKAs before the procedure (*Figure 3*).

### Anticoagulation management during electrophysiological studies and right-sided ablation procedures

**Figure 4:**
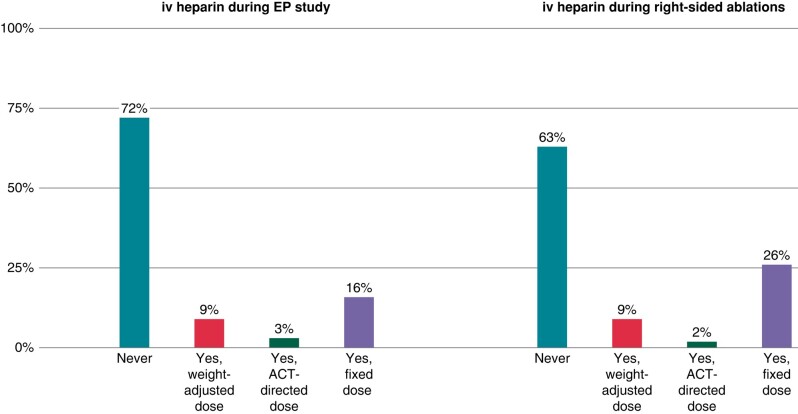
On the left—the graphic shows how many physicians administer iv heparin during EP studies; on the right—the picture illustrates how many operators administer iv heparin during right-sided ablations. EP, electrophysiological.

The majority of respondents (72%) never administers iv heparin during EP studies (*Figure 4*); 16% administer a fixed dose of iv heparin, 9% a weight-adjusted dose of iv heparin, and the remaining 3% an ACT-directed dose of iv heparin. Similarly, most of the respondents (63%) do not administer iv heparin during right-sided supraventricular tachycardia ablations (*Figure 4*); the remaining physicians use a fixed dose of iv heparin (26%), a weight-adjusted dose of iv heparin (9%), or ACT-directed dose of iv heparin (2%).

Of note, the majority of participants (69%) irrigates long sheath introducers during EP studies and right-sided ablation procedures; among them, 80% irrigate with heparin saline solution, and the remaining 20% use a normal saline infusion.

### Femoral venous access and post-procedural management

After the procedures, bed rest is recommended for <4 h by 12% of respondents, for 4–6 h by 70% of respondents, and for >6 h by 18% of respondents. Participants were also asked about the type of venous access site closure and management: manual compression was declared as the most common way to achieve the venous access closure (59%); Z-suture was preferred by 39% of respondents. Vascular closure devices (Perclose Proglide^TM^ and Angio-Seal) are used by 2% of participants. Among respondents who apply Z-suture in the femoral venous access, 7% of them remove it within 4 h, 14% of them 4–6 h later, 9% of them cut the suture 6 h later on the same day, and most of them (70%) remove the Z-suture the day after or even later.

Of note, compression dressings are routinely applied after the procedure by 25% of respondents. Among the latter, 14% remove the compression dressing within 4 h, 24% keep the dressing for 4 h, 42% for 6 h, and 20% for >6 h.

Finally, nearly half of respondents (46%) routinely remove the venous sheaths while aspirating blood with a syringe to pull out possible clots or thrombi.

Patients are commonly discharged at home on the day of the diagnostic EP study by 36% of respondents (53% of respondents never discharge the same day). The remaining 11% of them discharge the patient on the same day only if the procedure is performed in the morning. In case of atrioventricular nodal reentry tachycardia ablation, 74% of respondents never discharge patients on the same day; on the other hand, 17% of physicians always discharge in the evening after the procedure, and a further 9% of respondents discharge the patient on the same day only if the procedure is performed in the morning. Similarly, with right-sided accessory pathway ablation, 75% of respondents never discharge patients on the same day; on the contrary, 15% of respondents always discharge in the evening after the procedure, and the remaining 10% discharge only if the procedure is performed in the morning.

In case of right atrial tachycardia and right atrial flutter ablations, 77 and 75%, respectively, of respondents never discharge patients on the same day. When ablation of premature ventricular contraction or ventricular tachycardia in the right ventricle is performed, 82% of respondents never discharge patients on the same day; conversely, only 7% of respondents always discharge in the evening after the procedure.

### Venous thromboembolism prevention strategies after electrophysiological studies and right-sided ablation procedures

**Figure 5:**
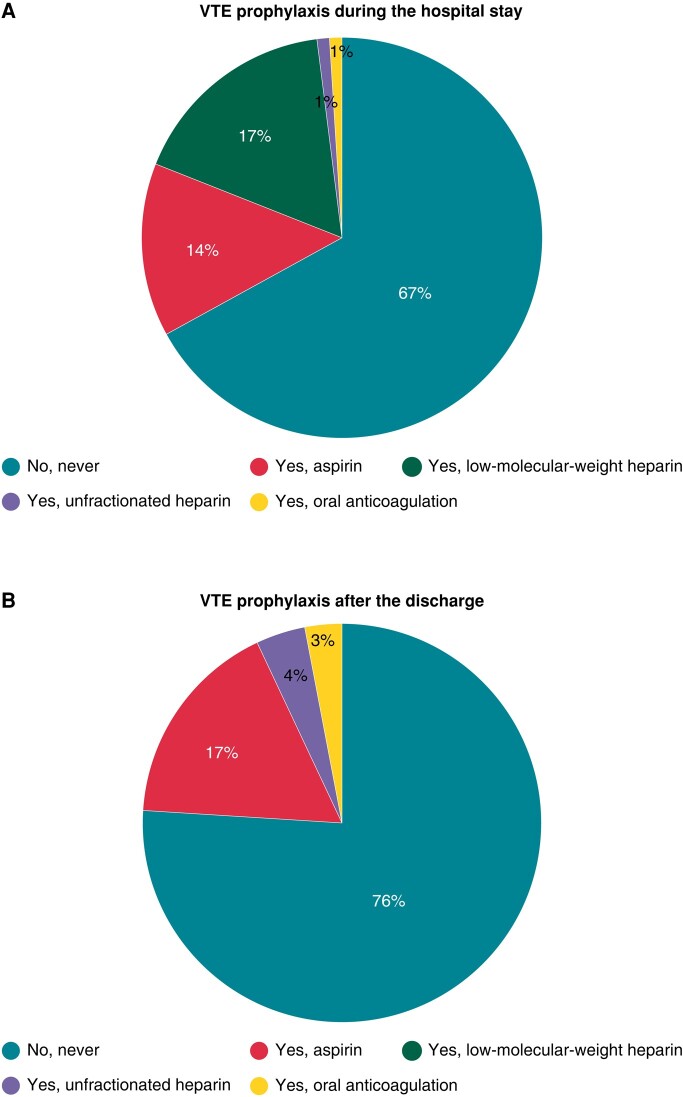
(*A*) The pie chart shows the proportion of physicians who gives VTE prophylaxis during the hospital stay and the type of medical prophylaxis. (*B*) The pie chart illustrated the rate of physicians who commonly prescribes VTE prophylaxis after the discharge and the type of medical prophylaxis. VTE, venous thromboembolism.

As shown in *Figure 5A*, medical prophylaxis for VTE following EP studies and right-sided ablations, during the hospital stay, is never done by two-third of physicians (67%); on the other hand, 17% of respondents routinely prescribe low-molecular-weight heparin (LMWH) after the procedures and a further 14% routinely prescribe aspirin. After discharge, medical prophylaxis for VTE is never done by 76% of physicians; of note, 17% of respondents routinely prescribe aspirin after discharge, and a small minority usually prescribes LMWH and oral anticoagulation (4 and 3%, respectively; *Figure 5B*).

Overall, 33% of the respondents routinely prescribe VTE prophylaxis early after the EP studies and right-sided ablations, and 24% of the respondents commonly prescribe VTE prophylaxis after discharge. If a pharmacological prophylaxis for VTE is prescribed, the mean duration is for 29 ± 21 days (median 29.5 days, IQR 14–30 days). When aspirin is used as VTE prophylaxis, the mean duration of the therapy is for 32 ± 17 days (median 30 days, IQR 27; 30); medical prophylaxis with LMWH is prescribed for a median duration of 6 days (IQR 3–75).

Most of the physicians (69%) do not change their strategy to prevent VTE based on the presence of specific risk factors. However, 31% of respondents report to change their strategy to prevent VTE according to specific risk factors; the latter ones are known, previous episodes of VTE (40%), concomitant therapy with oral contraceptives (16%), obesity (12%), smoking habit (5%), female gender (5%), known thrombophilic disease, or hypercoagulable condition (5%).

Of note, 25 of 184 respondents (13%) experienced within the last year at least one DVT related to right-sided ablation or EP study. In addition, 9% (*n* = 16) of respondents also stated that they experienced within the last year at least one PE related to right-sided ablation or EP study.

The analysis of specific subgroups (Northern vs. Southern Europe, fully trained EPs vs. junior/senior fellows) did not show significant differences in terms of intra-procedural iv heparin and VTE prophylaxis. In particular, a virtual line was drawn throughout the Alps in order to divide Europe into Northern and Southern Europe. Between 129 respondents from Northern Europe and 115 respondents from Southern Europe, no differences were found in terms of administration of iv heparin during EP studies (33 vs. 23%, respectively; *P* = 0.1) and right-sided ablations (39 vs. 34%; *P* = 0.4). No differences were also found in terms of VTE prophylaxis: 36% of participants from Northern Europe vs. 31% of participants from Southern Europe routinely prescribe VTE prophylaxis after the procedure (*P* = 0.4). Similarly, VTE prophylaxis after discharge was commonly prescribed by 26% of respondents from Northern Europe compared with 23% of respondents from Southern Europe (*P* = 0.6). Between 178 fully trained EPs and 66 junior/senior fellows, no differences were found in terms of administration of iv heparin during EP studies (31 vs. 23%, respectively; *P* = 0.2) and right-sided ablations (42 vs. 29%; *P* = 0.1). No differences were also found in terms of VTE prophylaxis: 34% of fully trained EPs vs. 30% of junior/senior EP fellows routinely prescribe VTE prophylaxis after the procedure (*P* = 0.5). Similarly, VTE prophylaxis after discharge was commonly prescribed by 25% of respondents from Northern Europe compared with 21% of respondents from Southern Europe (*P* = 0.5).

## Discussion

The main findings of the present survey are listed in the *Table 1*.

**Table 1:** Key messages

The echo-guided approach for femoral venous access is routinely adopted in roughly one-third of respondents
The right groin is usually chosen as the only access site
Intravenous heparin is adopted by, 28 and 37% of participants, respectively, during EP studies and right-sided ablations
Before the procedure: in patients on VKA ‘heparin bridging’ is still standard of care in 29%, and DOACs are interrupted by 44% of respondents
After the procedure/in-hospital: 33% of participants routinely prescribe VTE prophylaxis after the EP studies and right-sided ablations (mostly LMWH, 17% and aspirin, 14%)
After hospital discharge: 25% of respondents commonly prescribe VTE prophylaxis even after the discharge (mostly aspirin) for a median duration of 30 days

EP, electrophysiological; VKA, vitamin K antagonist; DOAC, direct oral anticoagulant; VTE, venous thromboembolism; LMWH, low-molecular-weight heparin.

In the last three decades, invasive EP studies and catheter ablation have become standard of care in the diagnosis and treatment of cardiac arrhythmias.^16–18^ Although catheter ablation has a high yield-to-complication ratio, the occurrence of venous thromboembolic complications is not neglectable.

As reported by previous studies, the evidence of asymptomatic DVT following catheter ablation is high, ranging between 0 and 16%.^7^ Nevertheless, the incidence of symptomatic DVT is much lower (0.5–0.8%).^1,13,19^ Three different mechanisms can be identified for the development of DVT/PE: (i) venous stasis and inflammation at the puncture site; (ii) thrombosis due to radiofrequency ablation; and (iii) groin bandage and immobility after procedures leading to venous stasis.

The Multicenter European Radiofrequency Survey,^1^ the largest cohort including 4398 patients referred for catheter ablation of supraventricular or ventricular tachycardias, showed that DVTs were identified in 0.5%, and the risk of embolic events following right-sided procedures was extraordinarily uncommon (0.06%). However, the true rate of VTE is surely underestimated as a systematic search of thrombo-embolic complications is not routinely done after procedures; as a matter of fact, asymptomatic rate of VTE has been reported to be much higher.^11,12^

Chen *et al.*^11^ reported an incidence of 17.6% for the development of asymptomatic, non-occlusive DVTs after EP studies using multiple venous sheaths. Repeat ultrasonography at 1 week documented regression of the non-occlusive DVTs in 92% of cases. A similar incidence of asymptomatic thrombosis (20%) was reported by Tiroke *et al*.^12^ despite the use of iv heparin.

Currently, there are no guidelines or consensus documents about the management of EP studies and right-sided ablations in terms of anticoagulation and VTE prevention before, during, and after the procedures. Of note, this survey shows that iv heparin is never administered by the majority of operators during EP studies or right-sided ablations (72 and 63% of participants, respectively). A Canadian survey recently reported similar findings.^5^ Generally, most of centres do not administer intra-procedural iv heparin during EP studies or right-sided ablation procedures, probably because the thrombotic risk is deemed to be largely overcome by the haemorrhagic risk. However, although venous thrombo-embolic complications are infrequent, they might potentially be life-threatening such as in case of PE. As VTE prophylaxis after right-sided EP procedures, some operators prescribe aspirin (oral, 50–150 mg) for a period of l–3 months following catheter ablation; on the other hand, some physicians commonly use heparin during the procedure and a few days after to prevent VTE.^7^ Although a discrete amount of operators gives aspirin for VTE prevention after right-sided EP procedures, there is no clear evidence supporting aspiring for prevention of DVT and PE. Low-molecular-weight heparin represents the cornerstone for prevention of DVT and PE following major surgery and invasive procedures.^20^

However, a recent multi-centre, randomized, non-inferiority trial compared enoxaparin 30 mg twice daily with aspirin 81 mg twice daily, as thromboprophylaxis in 12 211 patients with either surgically treated extremity fractures or with any pelvic or acetabular fracture. This study showed that aspirin was non-inferior to LMWH in preventing death and was associated with a low incidences of DVT and PE and low 90-day mortality.^21^ On the other hand, a recent and large meta-analysis concluded that aspirin was inferior when compared with other anticoagulants in VTE-related orthopaedic major surgery.^22^ Therefore, the role of aspirin for VTE prevention after surgery and invasive procedures is controversial and needs more evidence.

The present survey shows that systematic prophylaxis for VTE following EP studies and right-sided ablations is never done—during the hospital stay—by two-thirds of physicians (67%) and—after the discharge—prophylaxis for VTE is not considered by three-quarters (76%) of respondents. Nearly a fifth of the overall respondents (17%) routinely prescribes aspirin after the discharge. This EHRA survey confirms lack of systematic DVT prophylaxis following right-sided EP procedures and ablations. Although the ultrasound-guided approach has been shown to significantly reduce groin complications compared with conventional ‘blind’ puncture technique,^23–25^ a considerable amount of electrophysiologists (28%) still never use echo colour Doppler to puncture femoral veins in EP studies and right-sided ablation and 35% use it only in selected cases.

In patients under DOAC before the procedure, this therapy is usually not interrupted by most of the operators (56%); in case of VKA, most of respondents did not suspend the therapy before the procedure (66%). Among one-third of respondents who usually stop VKAs, a minority (29%) is still replacing oral anticoagulation with heparin before the procedure (the so-called ‘bridging heparin therapy’). However, bridging with LMWH has been clearly shown to be associated with higher incidence of groin haematoma/bleeding compared with continuation of VKA in left-sided procedures and device implantation procedures.^26,27^ With the current evidence of a lower haemorrhagic complication rate with uninterrupted VKA therapy, the bridging strategy should be clearly abandoned also in EP and right-sided ablation procedures.

A very interesting strategy is commonly adopted by one-third of participants who tailor their behaviour according to the presence of specific risk factors such as previous episodes of VTE, concomitant therapy with oral contraceptives, obesity, smoking habit, female gender, and known hypercoagulable conditions. In addition, an accurate sheath management with frequent flushing or continuous irrigation, especially with heparin saline solution, might help to minimize the risk VTE.^10^ In our survey, more than two-thirds of participants (69%) commonly irrigate the femoral sheaths, and among them, 80% routinely use heparin saline solution.

In our survey, only 25% of respondents regularly used compression dressings, with a variable duration of application that commonly does not exceed 6 h (80% of respondents used to remove compression dressings no more than 6 h later). There was relative homogeneity among the prescribed duration of bed rest after EP procedures, with most of physicians (82%) recommending 6 h or less. A potential preventive strategy to avoid thrombo-embolic complications is the ‘same-day discharge’.^28,29^ The present survey showed that this strategy is predominantly used in case of diagnostic EP studies. As a matter of fact, an early mobilization and a quick resumption to the routine daily life may significantly reduce the blood flow stasis and potentially reduce procedure-related VTE complications.

Of note, 13% of participants stated they had experienced at least one DVT related to right-sided ablation or EP study within the last year in their Centre. Likewise, 9% of respondents declared at least one PE related to right-sided ablation or EP study within the last year. These data might be strongly limited by a possible overestimation due to the possibility of more respondents coming from the same Centre.

By the way, our data confirm that the incidence of DVT/PE is rather low.

Larger, multi-centric, prospective, and randomized studies are needed to identify which, if any, of these post-procedural factors may affect the development of post-procedural VTE.

### Limitations

The present survey has limitations attributed to target respondents and questionnaire design. The survey included a limited number of selected physicians, and participation was completely voluntary, therefore being prone to selection bias. Moreover, junior EP fellows are included in the present survey (18% of all respondents); as they might tend to follow or reproduce their Centre protocols or Senior colleagues’ behaviour, this might represent a potential limitation.

Being a physician-based survey, physicians belonging to same EP centres might have answered leading to a possible overestimation of some peri-procedural management strategies.

Italy and Germany represented ∼40% of the 33 involved European countries.

Local vascular complications have not been collected by the questionnaire.

## Conclusions

The present survey shows that only a minority of operators routinely gives intra-procedural iv heparin and prescribes VTE prophylaxis after right-sided EP procedures. Compared with left-sided procedures like AF ablation, there are no consistent systematic antithrombotic management strategies. Thus, additional studies and guidelines or consensus statements are needed in order to standardize VTE prevention strategies throughout EP centres.

## Supplementary Material

euad364_Supplementary_Data

## Data Availability

Not applicable.
