# Supplementary material for: Prevention of venous thromboembolism in right heart–sided electrophysiological procedures: results of an European Heart Rhythm Association survey
Source: Europace. 2023 Dec 13;26(1):euad364. doi: 10.1093/europace/euad364 (PMC10754160; doi:10.1093/europace/euad364)
Supplement: euad364_Supplementary_Data [file euad364_supplementary_data.docx]

**SURVEY**

**Prevention of venous thromboembolism (VTE) in right-sided ablations**

Femoral venous access is usually obtained for all right-sided ablation procedures and for electrophysiological studies. Limited data are available regarding venous thromboembolism (VTE), specifically deep vein thrombosis (DVT) and pulmonary embolism (PE) following right-sided ablations (AV nodal ablation, right accessory pathway ablation, right atrial tachycardia ablation, etc…) and electrophysiological studies. Potential preventive strategies are unclear. There is considerable heterogeneity among EP centres in the pre-, peri- and post-procedural management of these procedures.

- Sex
- Age
- Country
- Type of institution (University hospital, Non-university hospital, Private hospital, …)
- Professional level (fully trained senior EP, junior EP, EP fellow)
- Years of training, e.g. 0-30 years

1. How many catheters do you normally use for EPS or right-sided ablation procedures (indicate the number of catheters in the table):

|  | N° of catheters |
| --- | --- |
| EPS |  |
| AVNRT |  |
| AVRT |  |
| AT |  |
| AFL |  |
| AVN |  |
| PVC/VT |  |

1. Which groin is commonly used for venous access?

- Right groin
- Left groin
- Both groins

1. Do you use echo-colour-doppler for femoral venous access?

- Always
- Sometimes – in selected patients
- Never

1. Do you use jugular venous access to place the catheter in the coronary sinus?

- Yes, always
- No, never
- Yes, only in some procedures (please specify __________)

1. Do you regularly administer IV heparin during diagnostic EP studies?

- Never
- Fixed dose
- Weight-adjusted dose
- ACT-directed dose

1. Do you regularly administer IV heparin during SVT right-side ablations?

- Never
- Fixed dose
- Weight adjusted dose
- ACT-directed dose

1. Do you irrigate long sheath introducers?

- No
- Yes

If yes in question 7:

7a. What infusion do you use to irrigate long sheath introducers?

- Heparinised saline infusion
- Saline infusion

1. If the patient is using a direct oral anticoagulant, do you recommend suspending it before the procedure?

- No
- Yes

If yes in question 8:

8a. How long before the procedure do you stop direct oral anticoagulants?

- < 24 h
- 24-48 h
- > 48 h

If yes in question 8:

8b. When do you restart direct oral anticoagulants after the procedure?

- Same day
- Next day
- 2 days later
- ≥2 days later

1. If the patient is on a vitamin K antagonist, do you stop them before the procedure?

- No
- Yes

If yes in question 9:

9a. How long before the procedure do you stop the vitamin K antagonist?

- < 24 h
- 24-48 h
- > 48 h

If yes question 9:

9b. Do you bridge patients with low-molecular heparin when stopping vitamin K antagonists?

- No
- Yes

1. For how long do you order bed rest after EPS or ablation of right-sided procedures?

- < 4h
- 4-6h
- > 6h

1. What kind of venous access site closure/management do you use? (Select all that apply)

- Manual compression
- Z-suture
- Compression dressings
- Other (please specify)

11a. If you use Z-suture, when do you remove it?

- < 4h later
- 4-6h later
- Same day but >6h later
- Next morning or later

11b. If you use compression dressings, for how long is the compression dressing applied?

- < 4h
- 4 h
- 6 h
- > 6h

1. Do you usually remove the sheaths in aspiration at the end?

- Yes
- No

1. Do you routinely prescribe deep venous thrombosis prophylaxis after EPS or ablation procedures during a hospital stay?

- Yes, subcutaneous unfractionated heparin
- Yes, subcutaneous low molecular weight heparin
- Yes, aspirin
- Yes, oral anticoagulation
- No

1. Do you routinely prescribe deep venous thrombosis prophylaxis after EPS or ablation procedures after the discharge?

- Yes, subcutaneous unfractionated heparin
- Yes, subcutaneous low molecular weight heparin
- Yes, aspirin
- Yes, oral anticoagulation
- No

If yes in question 14:

14a. For how many days do you prescribe deep venous thrombosis prophylaxis?

1. Do you routinely discharge the patient home the same day if the procedure was performed without complications?

|  | Yes | No | Timing of discharge varies by operator | Timing of discharge varies by timing of the procedure (e.g. morning vs. afternoon) |
| --- | --- | --- | --- | --- |
| EPS |  |  |  |  |
| AVNRT |  |  |  |  |
| AVRT |  |  |  |  |
| AT |  |  |  |  |
| AFL |  |  |  |  |
| PVC/VT |  |  |  |  |

1. How many right-sided procedures were performed at your centre in the last 12 (or 24/36) months?
2. Within the last year, did any of your patients experience a thromboembolic complication (DVT/PE) possibly related to right-sided ablation of EPS?

- Yes, deep venous thrombosis How many?
- Yes, pulmonary embolism How many?
- No

1. At your centre, is an institutional reporting system used for the collection and assessment of complications of EP procedures?

- No
- Yes

1. Do you change your strategy to prevent thromboembolism based on the presence of specific risk factors? (such as obesity, previous PE, contraceptives, gender, smoking habit, etc.)

- No, never
- Yes (please specify the risk factors you take into account ______________________)
